# Impotent Oxford and Its Melancholy Hospital

**Published:** 1892-05-14

**Authors:** 


### The Hospital

An Institutional Journal of

Science, Medicine, Nursing, and Philanthropy.

*For Hospitals, Asylums, Infirmaries, Dispensaries, Medical Practitioners, Students, Nurses, and the Charitable Public.*

Vol. XII.—No. 294.] [May 14, 1892.

### Impotent Oxford and its Melancholy Hospital.

A little less than a year ago Oxford needed fifteen thousand pounds, wherewith to bring up its one infirmary to the level of modern sanitary requirements and practical efficiency. It issued an appeal for ten thousand, but so far the response to its call has only yielded about fifteen hundred. What do some of the managers think to be the proper steps to take under these discouraging circumstances? Do they put better arguments before their constituents, and appeal to the benevolent with more energy and enthusiasm? Nothing of the kind! Instead of that, they turn round upon the newspaper that pointed out the sanitary and other defects of their institution, and abuse it with might and main. They even hint at legal proceedings. There is as much logic and honest English sense in this proceeding as there would be in that of a drowning man who should roundly abuse a passing friend that happened to pull him out of the water.

Nothing gives one a clearer insight into human nature than the behaviour of the same men under different sets of circumstances. The Rev. Dr. Bright, the president of the Governors' Board, is, as everybody knows, also Master of University College. If Dr. Bright were to see one of the members of his own college falling into heedless slovenliness and unwholesomeness of life, his first instinct would be to point out to him the folly and danger of his ways, and to set before him some counsels of academic perfection. Similarly, if any of the physicians or surgeons of the infirmary were to see a patient going backwards towards deeper disease and danger instead of forward towards health, the very first step taken would be to find out the cause of the retrogression, to remove it, and to apply such therapeutic stimulus to the patient's failing powers, as would energetically push him forward again towards the goal of perfect well-being.

But other circumstances, other methods of behaviour. Dr. Bright and his fellow-managers are the responsible heads of an institution that has fallen behind the times; a hospital whose sanitation is defective, its appearance melancholy and depressing, and its general capacity for the work of a hospital that of thirty years ago rather than of to-day. Under these circumstances it was clearly the duty of somebody to strive to raise the sinking fortunes of an absolutely necessary institution. This journal, which exists in the interests alone of medicine and medical charity in the largest sense, felt bound to put forth a helping hand when a responsible representative saw an important hospital falling to a third rate level of everything that constitutes medical and sanitary excellence. That representative did, indeed, exactly what Dr. Bright himself would have done in similar circumstances.

Is the Oxford Infirmary of average excellence in construction and sanitation, or is it not? That is the only practical point at issue. If it be, why did the managers appeal to their constituents for ten thousand pounds wherewith to improve it? But if it be not, where is the justice and reasonableness of abusing a friendly critic who makes a much-needed comparison between the Radcliffe Infirmary and hundreds of similar institutions which he has visited and inspected in practically every part of the civilised world? The present writer, who claims to be an independent umpire between the parties to the controversy, holds that instead of abuse our "Special Commissioner" has earned the gratitude of the Oxford managers. He has done for them what probably not one of themselves could have done, namely, instituted an expert's comparison between the Radcliffe Infirmary and most of the best hospitals that have been built within what may be called the modern period. Is it, or is it not the case, that John Howard actually condemned this very infirmary not less than a hundred years ago? If the Radcliffe Infirmary of the eighteenth century did not show favourably in comparison with the hospitals and lazarettos of the Mediterranean towns and cities of that period, is it so very surprising that when compared with the noble institutions of England and America it should now appear out of date, and unworthy of the name and fame of Oxford?

The Governors, at their meeting, held a week or two ago, discussed the propriety of calling in a Balaam to anathematise those whom they considered their enemies. We do not care to say quite what we think about the childish inconsequence of the motive which animated the propounder of this proposal. But to the proposal itself we give our unqualified approval. Sir Douglas Galton was the Cæsar to whom appeal was to be made. The real friends of the Oxford Infirmary, and of hospital excellence in general, could wish no better choice. By all means let Sir Douglas Galton be called in: and when he has pronounced, as he will pronounce, his friendly and rational verdict, let the people of Oxford shake off their unworthy lethargy and rise to the level of their public duty like strong and honest Englishmen.

Dr. Bright and his fellow Governors, notwithstanding that some of them insist upon regarding us as foes, have our entire sympathy. The people of Oxford on the other hand have merited the reprobation of every philanthropic sanitarian. The authorities, in a spirit of loyalty to truth, admitted the justice of our criticism, though they did not forgive the critic. But at least they went a long way in the direction he pointed out, and asked their fellow citizens to give them ten thousand pounds to carry out the much-needed work. The gentlemen of Oxford and the county, the rich men of business who grow fat and sleek under the generous shelter of the University, and the members of the most splendid of all the world's colleges, have responded with a miserable fifteen hundred pounds, and have taken little short of a year to screw their courage to that disgraceful sticking place. The business element of Oxford, with the exception of a few large subscriptions, seems to have done absolutely nothing at all.

Now there is no escape from one of these two positions: either Oxford must bring up her infirmary to the sanitary and generally wholesome requirements of the time, or she must accept the unenviable distinction of being the first in culture and the last in charity of all the cities of the world. Such a position ought to be intolerable to the men of Oxford; and for our part, we are resolved that nothing shall be left undone to make it intolerable. But we by no means wish to apply whips and spurs to the sides of good intent. There can be no real difficulty in securing all the money that is required for the construction of a perfect and perfectly equipped infirmary if the work is set about with the requisite wisdom and zeal. The very first thing to be done should be to call the city and the county together at a public meeting and to appoint a special Buildings Committee, with a special secretary and full powers for this one purpose. That committee, which need not be large, which had better in fact be small, should consist of persons of energy and enthusiasm, whose sole business it should be to raise the necessary funds. The sum of fifteen thousand pounds should be asked for, and no less; and the purpose of every official, great and small, connected with the institution should be to raise at beautiful Oxford a hospital in every way worthy of the University, the city, and the noble medical science and charity of the times.

It has been pointed out in these pages before that Oxford puts a crown of shame upon her head when she refuses to listen to the demands of the science and the brotherly kindness which represent the Time-spirit of the age. The very vital breath of the close of the century is a large, generous, and scientifically instructed humanitarianism. The days of mean and miserable makeshifts in medical science and charity are gone, we trust, for ever. But especially ought they to be gone from Oxford, of all places in the world. Dr. Bright, who personally has worked with admirable zeal and energy, must once more raise his flag aloft; he must call the people of the town and of the county together; and the members of the University must rally to his standard. If the subject be properly placed before the thousands of prosperous Englishmen who dwell within a radius of twenty miles of the Radcliffe Infirmary, it is impossible but that the needed money shall be subscribed, even three or four times over. The beautiful charity of medicine must be provided with a beautiful home at Oxford; and the new science and art of healing must be lodged in an abode worthy of the new time. As was said on an unprecedented occasion many hundreds of years ago—if we "should consent to hold our peace, the very stones of which the melancholy infirmary is built would begin to cry out."

